# Development and identification of four new synthetic hexaploid wheat lines with solid stems

**DOI:** 10.1038/s41598-022-08866-x

**Published:** 2022-03-22

**Authors:** Dongyu Liang, Minghu Zhang, Xin Liu, Hui Li, Zhenjiao Jia, Dinghao Wang, Ting Peng, Ming Hao, Dengcai Liu, Bo Jiang, Lin Huang, Shunzong Ning, Zhongwei Yuan, Xuejiao Chen, Lianquan Zhang

**Affiliations:** 1 State Key Laboratory of Crop Gene Exploration and Utilization in Southwest China, Sichuan Agricultural University, Wenjiang, Chengdu, 611130 China; 2 Triticeae Research Institute, Sichuan Agricultural University, 211 Huimin Road, Wenjiang, Chengdu, 611130 China

**Keywords:** Plant sciences, Plant breeding

## Abstract

Stem solidness is an important agronomic trait for increasing the ability of wheat to resist lodging. In this study, four new synthetic hexaploid wheat with solid stems were developed from natural chromosome doubling of F_1_ hybrids between a solid-stemmed durum wheat (*Triticum turgidum* ssp. *durum*, 2n = 4x = 28, AABB) and four *Aegilops tauschii* (2n = 2x = 14, DD) accessions. The solid expression of the second internode at the base of the stem was stable for two synthetic hexalpoid wheat Syn-SAU-117 and Syn-SAU-119 grown in both the greenhouse and field. The lodging resistance of four synthetic solid-stem wheats is stronger than that of CS, and Syn-SAU-116 has the strongest lodging resistance, followed by Syn-SAU-119. The paraffin sections of the second internode showed that four synthetic wheat lines had large outer diameters, well-developed mechanical tissues, large number of vascular bundles, and similar anatomical characteristics with solid-stemmed durum wheat. The chromosomal composition of four synthetic hexaploid wheat was identified by FISH (fluorescence in situ hybridization) using Oligo-pSc119.2-1 and Oligo-pTa535-1. At adult stage, all four synthetic hexaploid wheat showed high resistance to mixed physiological races of stripe rust pathogen (CYR31, CYR32, CYR33, CYR34). These synthetic hexaploid wheat lines provide new materials for the improvement of common wheat.

## Introduction

Lodging, defined as the permanent displacement of stems from the vertical direction, is caused by a loss of balance within the body of the plant and can reduce the grain yield of wheat by 12–80%^1–4^. Wheat lodging includes stem lodging and root lodging^5^. Commonly, lodging in wheat occurs as a result of stem lodging rather than root lodging^6^. Stem lodging is the bending or breakage of the stem base caused by stem mechanical failure^5^. The lodging resistance of wheat stems is the result of the synergistic effect of the morphological characteristics and anatomical structures of wheat^7^. Previous efforts to reduce the occurrence of lodging in wheat have focused on reducing the height of plants and the use of plant growth regulators^8^. Another potential strategy is to breed wheat varieties with stems that have increased mechanical strength^9–11^. Therefore, improving the strength of wheat stems is an ideal way to increase the ability of wheat to resist lodging^12^.

Previous studies have shown that the second internode at the base of the wheat stem plays a vital role in enhancing lodging resistance^13,14^. Increasing the outer diameter of the wheat stalk or thickening the stem wall at the base of the wheat could greatly improve lodging resistance^15,16^. A larger mechanical structure and thicker parenchyma, more vascular bundles and a larger vascular bundle area are also conducive to improving lodging resistance^12,17^. The ratio of the stem wall thickness to the outer stem diameter and the mechanical tissue contents of solid-stemmed wheat are significantly higher than those of common wheat^12,13,18,19^. Therefore, solid-stemmed wheat has higher stalk strength and stronger lodging resistance than common wheat^12,13^.

There are three sources of stem solidness in common wheat (*Triticum aestivum* L.). (1) S-615: a solid-stemmed landrace from Portugal^18^, (2) Conan: a semisolid-stemmed hard red spring wheat, developed by WestBred, LLC, USA^20^, and (3) Janz: a solid-stemmed white spring wheat that derives its stem solidness from *Agropyron elongatum*^19^. The most common wheat cultivars in North America derived their stem solidness from the Portuguese landrace S-615^18^, with the genes influencing stem solidness localized to chromosomes 3B, 3D, 5A, 5B and 5D. The major QTL designated *Qss.msub-3BL* has been reported to be associated with the solid-stem trait, contributing up to 76% of the total genetic variation for stem solidness^21^. Under the influence of *Qss.msub-3BL*, early stem solidness was expressed during both jointing and booting, and late stem solidness was expressed after anthesis^22^. One differentially expressed gene, *TraesCS3B01G608800* (KAF7034036.1), was present as a single copy in IWGSC RefSeq v1.0 but showed copy number variation associated with stem solidness in a diverse panel of hexaploid cultivars^23^.

Durum wheat (*Triticum turgidum* L. ssp. *durum*, AABB, 2n = 4X = 28) includes an abundance of solid-stemmed varieties, landraces and old varieties^24^. Currently, there are at least two main sources of stem solidness in durum wheat: (1) Golden Ball: a solid-stemmed durum cultivar from South Africa^25^ and (2) Biodur (Valdur//Wascana/Durtal): a solid-stemmed durum cultivar from Germany^26^. Currently, the solid-stemmed durum cultivars registered for use in western Canada, CDC Fortitude, AAC Raymore, and AAC Cabri, all derive their stem solidness from the German cultivar Biodur^27–29^. In durum, a single dominant gene designated *SSt1* confers the solid-stemmed phenotype and had been mapped to chromosome 3BL in the region of the *Qss.msub-3BL* locus^30–32^. The two sources of stem solidness in durum wheat (Golden Ball and Biodur) were different in haplotype around *SSt1,* although this QTL had been mapped to 3B in both sources^32^. The synergistic two-way interaction between *SSt1* and other secondary QTLs on the chromosome resulted in a higher rate of solid stems than when *SSt1* was used alone^32^. The solidity of the stem was complementary to many factors^33^, and the additive effect of the *SSt1* resistance allele in durum wheat produced stem solidness three times that of common wheat, with an additive effect^32,33^. *TRITD3Bv1G280530* (LOC123067038)in solid-stemmed and hollow-stemmed durum wheat differed in copy number and was most likely to be a candidate gene in the *SSt1* interval^34^.

Durum wheat had greater stem solidness and was genetically more stable than common wheat cultivars^26,35,36^. Crossing with bread wheat directly or crossing with the diploid *Ae. tauschii* Coss. to develop synthetic hexaploid wheat are two alternative methods to utilize durum wheat genetic resources^37^. Efforts began in the 1940s to transfer solid stems from Golden Ball to hexaploid wheat by direct crossing, but solidness was suppressed, and only hollow-stemmed offspring were produced^18,35,38,39^. This suppression was overcome by crossing Golden Ball with *Ae. squarrosa* L. to create a synthetic hexaploid (P89-77-1F_4_), which expressed pith in the culm lumen. The offspring of P89-77-1F_4_ were backcrossed to the hollow-stemmed hexaploid wheat cultivar AC Elsa^40^, and then two solid-stemmed hexaploid spring wheat lines (PI 633,737 and PI 633,738) were developed and released^41^. However, both lines were still taller and matured later than AC Elsa, which averaged 95 cm in height and reached maturity in 104 d in the brown soil zones and in 107 d in the dark brown soil zones^40,41^.

Given the consistent expression of solid stems in durum wheat, it is necessary to transfer solid stems from more durum wheat lines to common wheat lines. However, there are few reports on the transfer of stem solidness from durum wheat to common wheat^26,41^. Moreover, research on the expression of stem solidness of durum wheat in synthetic hexaploid wheat is limited. In this study, four synthetic hexaploid wheat lines were developed from the cross of semidwarf solid-stemmed durum wheat and four different *Ae. tauschii* accessions. Furthermore, these synthetic hexaploid wheat plants were identified by cytological identification, observation of the solidity and anatomical structure of the second internode at the base of the stem, and determination of lodging resistance.

## Materials and methods

### Plant materials

One solid-stemmed durum wheat (*T. turgidum* ssp. *durum*, 2n = 2x = 28, AABB) Ma and four different *Ae. tauschii* ssp. *tauschii* (2n = 2x = 14, DD) accessions AS78, AS92, AS95, and AS96 were used in this study. The common wheat line SY95-71 was used as a susceptible control in stripe rust resistance analysis, and Chinese spring (CS) was used as a hollow-stemmed control. The durum wheat Ma is a semidwarf durum wheat with a plant height of approximately 80 cm that was kindly provided by George Fedak of the Ottawa Research and Development Centre in Canada. The lines with the code AS were kept in our institute. All germplasm materials generated and analyzed from this research have been stored in the Triticeae Research Institute, Sichuan Agricultural University. These materials can be shared with researchers for academic purposes upon request to the corresponding authors. Experimental research and field studies on the plants in our study, including the collection of plant material, comply with relevant institutional, national, and international guidelines and legislation.

### Hybridization and natural chromosome doubling

Crosses were made using *T. turgidum* ssp. *durum* Ma as the female parent and *Ae. tauschii* AS78, AS92, AS95, and AS96 as the male parents in the field in the 2017–2018 wheat growing season. Emasculation and pollination were performed following Ref^42^. No embryo rescue or hormone treatment was applied for the production of F_1_ seeds. F_1_ seeds were germinated in Petri dishes, and the root tips were analyzed cytologically. Then, F_1_ hybrid plants were transplanted to the field (at Wenjiang Experimental Station of Sichuan Agricultural University, 30°36′ N, 103°41′ W) during the 2018–2019 wheat growing season. F_1_ plants were self-fertilized through natural chromosome doubling, and the seed set ratios (percentage of selfed seed set per self-pollinated floret) for each plant were calculated.

### Agronomic trait comparisons

The newly developed synthetic hexaploid wheat and its parents were sown in the field in October 2019. Individual plants were grown 10 cm apart within rows, with 30 cm between rows, which were 1.5 m long. Each line was planted in two rows. Plant height, the tiller number per plant, spike length, and seed setting were observed following Ref.^43^. Data from 10 plants were used to compare trait differences with the *t* test.

### Stripe rust resistance evaluation

Field evaluation of stripe rust resistance was conducted at the adult stages during the 2019–2020 crop seasons. The lines were grown as individual plants spaced 10 cm apart within rows, with 30 cm between rows, which were 1 m in length. The highly susceptible stripe rust spreader variety of wheat SY95-71 was planted on both sides of each experimental row. Six weeks after planting, seedlings were inoculated with a mixed population of Chinese *Puccinia striiformis* f. sp. *tritici* (*PST*) races CYR31, CYR32, CYR33, CYR34. The stripe rust infection type was recorded three times at 10-day intervals. Disease notes were taken when the flag leaves of SY95-71 showed full susceptibility. For each plant, the infection type (IT) was recorded on a scale of 1–9^44^. The *PST* responses were recorded as resistant (1–2, highly resistant; 3, resistant; 4, moderately resistant), intermediate (5), or susceptible (6–7, moderately susceptible; 8, susceptible; 9, highly susceptible).

### Stem solidity identification

The expression of solid-stemmed traits was evaluated in the four synthetic hexaploid wheat plants, Ma and CS, which were planted in the greenhouse in July 2020 and in the field in October 2020, respectively. The stems were sampled following Ref.^12^. More than ten stems from the main tiller were randomly selected after flowering and were cross-sectionally cut at the center of each internode. The level of stem solidity was rated as 1–5 (1 for hollow and 5 for solid) following Ref.^45^.

### Lodging resistance identification

The breaking resistance and bending moment of the second internode of Ma, synthetic wheat and CS were measured in the field^46^, and the lodging index was calculated following Ref.^47^. At 30 days after heading, the main stems were selected, and the internodes were numbered 1–5 consecutively from the bottom to the top of the stem. The fresh weight from the base of every internode to the top of the spike and the length from the base of the internode to the top of the spike were measured. The bending moment = the length from the base of the internode to the top of the spike × the fresh weight from the base of this internode to the top of the spike. Then, the length of the second internode was measured, and the midpoint was determined. Then, 5 cm of stalk at both ends of the midpoint was retained, and the extra parts were removed. The second internodes were placed horizontally to fix their ends, a stalk strength measuring instrument (YYD-1A, China) was placed vertically at the midpoint, and force was slowly applied to break the stems. The magnitude of the force is the internode breaking resistance. Finally, the lodging index (bending moment/breaking resistance × 100) was calculated. Data from the second internode for each line were used to compare trait differences with the *t* test.

### Observation of the anatomical structures of stems

The internodes were numbered consecutively from the base to the top of the stem. At the flowering stage, the main tiller was selected. The center of the second internode of the wheat stem base was cut into 1 cm pieces and then soaked in FAA fixative for more than 24 h following Ref.^13^. The samples were sent to Wuhan Servicebio Biological Technology Co., Ltd. for preparation of paraffin sections. CaseViewer 2.3 (https://www.3dhistech.com/solutions/caseviewer/, 3DHISTECH, Hungary) was used to view the results of the paraffin section analysis. The diameters of the stem and medullary cavity and the thickness of the mechanical tissue were measured. The number of vascular bundles was calculated. Each sample was measured 25 times, and the average value was taken. Trait differences were compared with the *t* test.

### Cytological observations

Cytological observations were made for the number of chromosomes of root tip cells and chromosome pairing of pollen mother cells (PMCs) following Ref.^42^. Multicolor fluorescence in situ hybridization (FISH) was performed on the root tip cells of the plants with 2n = 42 using oligonucleotide probes Oligo-pSc119.2-1 and Oligo-pTa535-1 following Ref.^48^. For meiotic analysis, at least 30 PMCs were observed for each line. Univalents (I) and bivalents (II) were counted, and their average numbers were calculated. All probes were synthesized and labeled with FAM or Tamra (TSINGKE Biological Technology Company, Chengdu, China). Hybridization signals were observed using an Olympus BX-63 epifluorescence microscope, and the images were photographed using a Photometric SenSys Olympus DP70 CCD camera (Olympus, Tokyo). Raw images were processed using Photoshop CS6 (Adobe Systems Incorporated, San Jose, CA, USA). Individual chromosomes of synthetic hexaploid wheat were compared with the karyotypes of the previously published FISH patterns of newly synthesized hexaploid wheat lines^49^.

## Results

### Development of four synthetic hexaploid wheat lines

**Figure 1:**
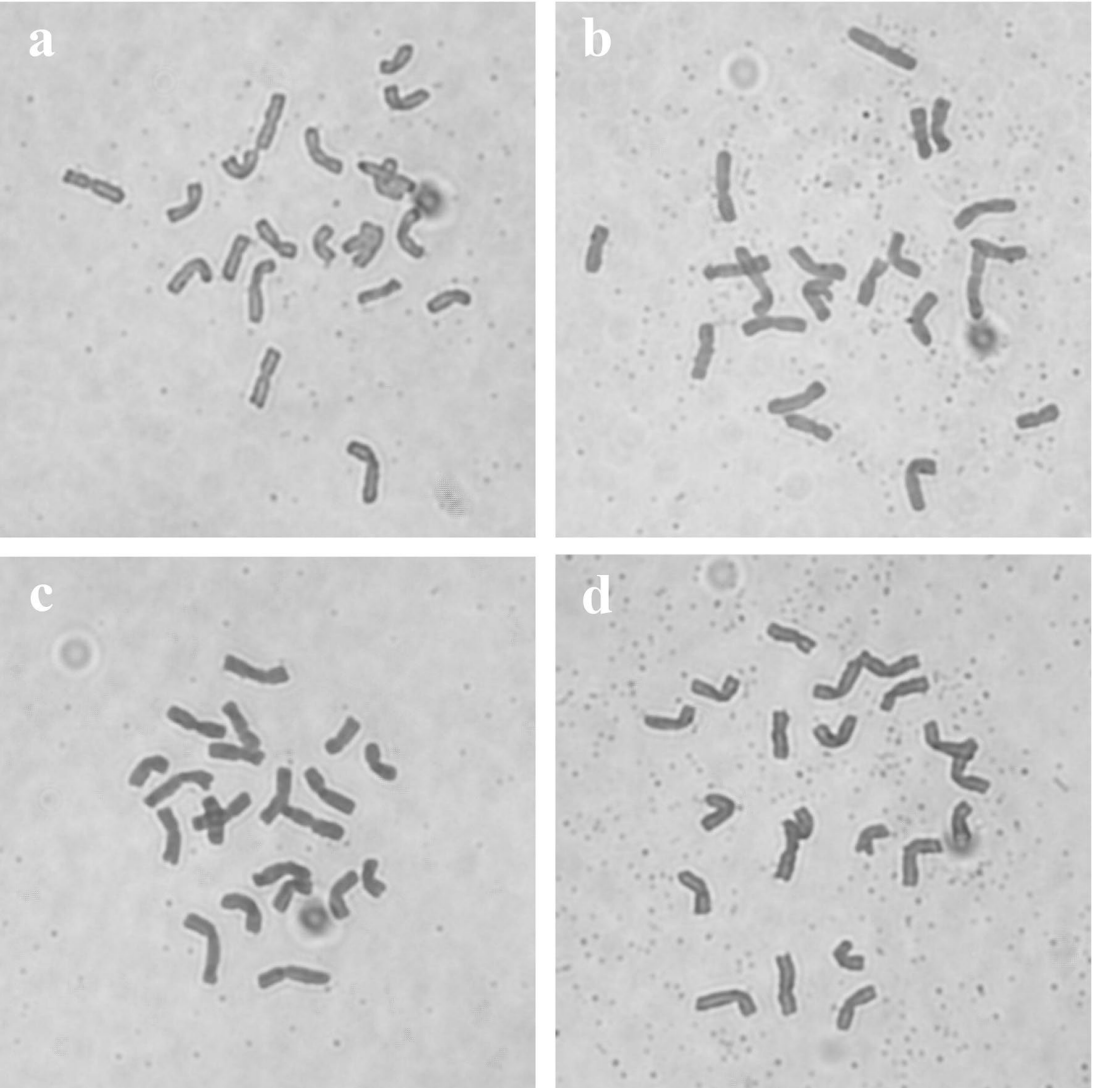
Root tip chromosomes of the F_1_ hybrid of Ma (*Triticum turgidum* ssp. *durum*) and *Aegilops tauschii* Cosson. (**a**): Ma/AS78 F_1_; (**b**): Ma/AS92 F_1_; (**c**): Ma/AS95 F_1_; (**d**): Ma/AS96 F_1_.

Four F_1_ hybrid combinations were obtained from crosses between solid-stemmed Ma as the female parent and four different *Ae. tauschii* accessions AS78, AS92, AS95, and AS96 as the male parents in 2018. The true F_1_ hybrids were found cytologically to have a chromosome number of 21 (Fig. 1). The plant height and spike length of the F_1_ hybrids were similar to those of their female parent, but the number of tillers reached 10–20, which was similar to that of their male parent. The selfed seed set rates of F_1_ hybrid combinations were 16.71%, 16.48%, 20.36% and 25.77% for Ma/AS78, Ma/AS92, Ma/AS95, and Ma/AS96, respectively (Table 1). Then, four newly synthetic hexaploid wheat lines were developed from natural chromosome doubling of these true F_1_ hybrids, coded by Syn-SAU-116, Syn-SAU-117, Syn-SAU-118, and Syn-SAU-119.

**Table 1:** Self seed setting rate of hybrid F_1_ between Ma and different *Ae. tauschii* accessions.

Hybrid combination	No. selfed florets	No. self-setting seeds	Seed setting (%)
Ma/AS78 F_1_	700	117	16.71
Ma/AS92 F_1_	910	150	16.48
Ma/AS95 F_1_	722	147	20.36
Ma/AS96 F_1_	1230	317	25.77

### Agronomic traits of the four synthetic hexaploid wheat lines grown in the field

**Figure 2:**
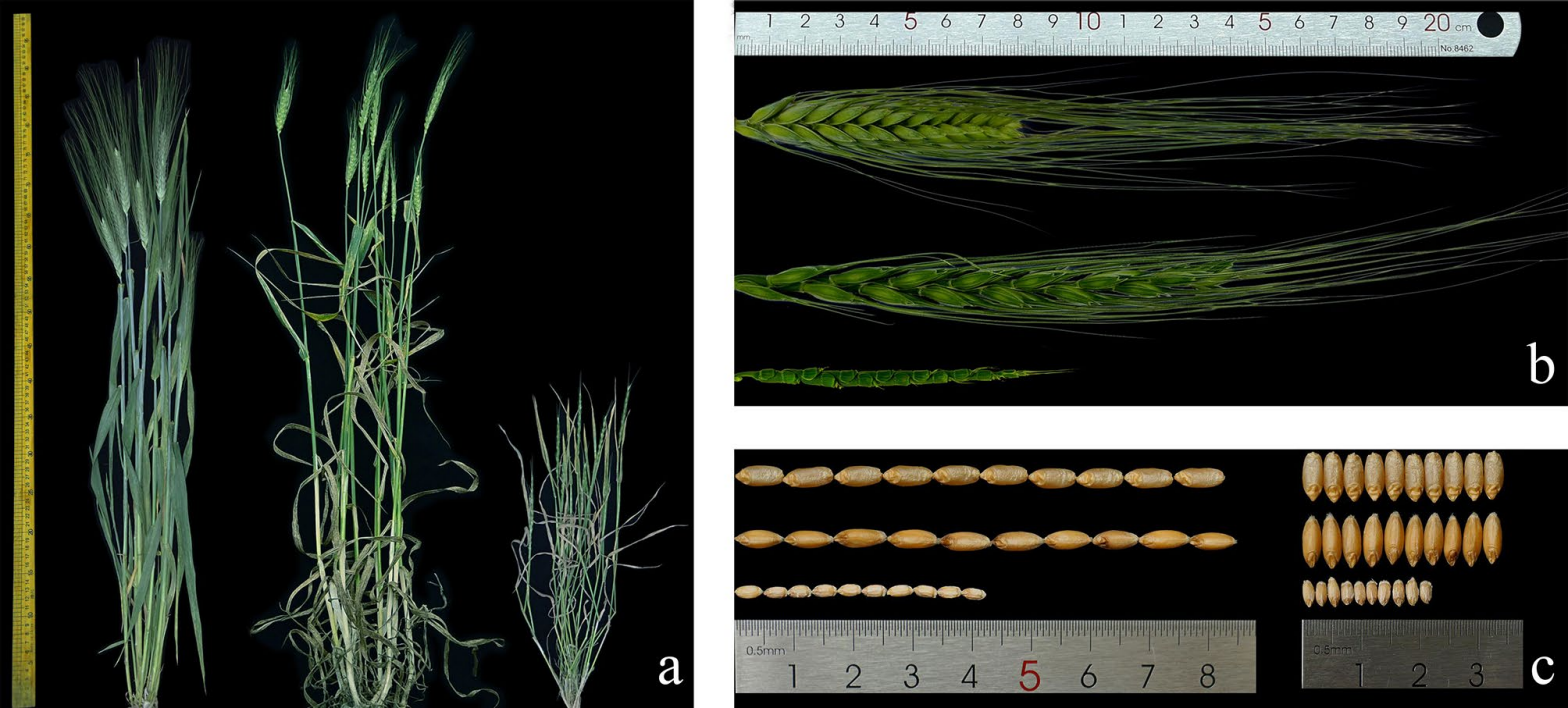
Morphology of synthetic hexaploid wheat and its parents. (**a**): Ma (left), Syn-SAU-119 (middle) and AS96 (right); (**b**), (**c**): Ma (top), Syn-SAU-119 (middle) and AS96 (bottom).

**Figure 3:**
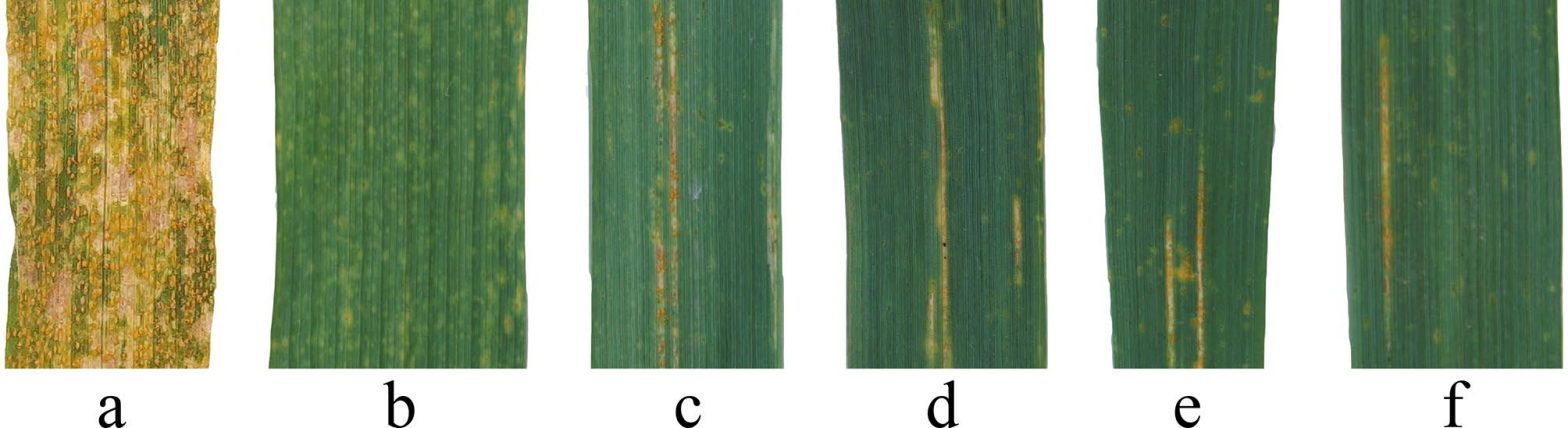
Stripe rust resistance of synthetic wheat at the adult stage. (**a**): SY95-71; (**b**): Ma; (**c**): Syn-SAU-116; (**d**): Syn-SAU-117; (**e**): Syn-SAU-118; (**f**): Syn-SAU-119.

The agronomic traits of the four synthetic hexaploid wheat varieties and their parents were evaluated in the field (Fig. 2, Table 2). The plant heights of all four synthetic wheat plants were higher than those of their parents (Fig. 2a), and there were very significant differences from their male parents. The plant heights of Syn-SAU-117 and Syn-SAU-119 were significantly different from those of their female parents. The spike lengths of the four synthetic wheat plants were longer than those of their parents, and there were very significant differences from those of their parents (Fig. 2b). The seed length and width were similar to those of Ma but not as full as Ma (Fig. 2c). The synthetic wheat lines Syn-SAU-116, Syn-SAU-118 and Syn-SAU-119 had a higher self-seed setting rate, and Syn-SAU-117 had a lower self-seed setting rate. At the adult stage, all four newly synthetic hexaploid wheat lines were resistant to stripe rust (IT, 2–3) (Fig. 3), Ma was highly resistant (IT, 1) (Fig. 3), and all four *Ae. tauschii* accessions AS78, AS92, AS95, and AS96 were susceptible (IT, 7–8) according to Ref.^50^.

**Table 2:** Agronomic trait comparison of synthetic hexalpoid wheat and their parents.

Plant materials	Plant height (cm)	Spike length (cm)	Seed setting (%)	Solidness (field/greenhouse)	Adult ITs^a^
Ma	82.3	10.46	54.6	5.0/5.0	1
Syn-SAU-116	90.2^##^	14.4**^##^	81.87**^##^	5.0/4.2	3
AS78	50	8.06	70.69	1.3/-^b^	7
Syn-SAU-117	92*^##^	13.6**^##^	47.37^#^	4.1/3.2	2
AS92	61.7	9.39	78.13	1.2/-^b^	8
Syn-SAU-118	88.25^##^	14.5**^##^	76.77*	4.5/5.0	2
AS95	50.4	8.54	68.32	1.0/-^b^	7
Syn-SAU-119	91.5*^##^	14.28**^##^	70.51	5.0/5.0	2
AS96	48.4	8.01	73.16	2.0/-^b^	7

^a^the infection type to stripe rust; ^b^no data.

*Significantly different from *T. durum* Ma at the 0.05 level, **at the 0.01 level; ^#^ significantly different from *Ae. tauschii* at the 0.05 level, ^##^at the 0.01 level.

### Observation of the stem solidity of synthetic hexaploid wheat lines grown in the greenhouse and field

**Figure 4:**
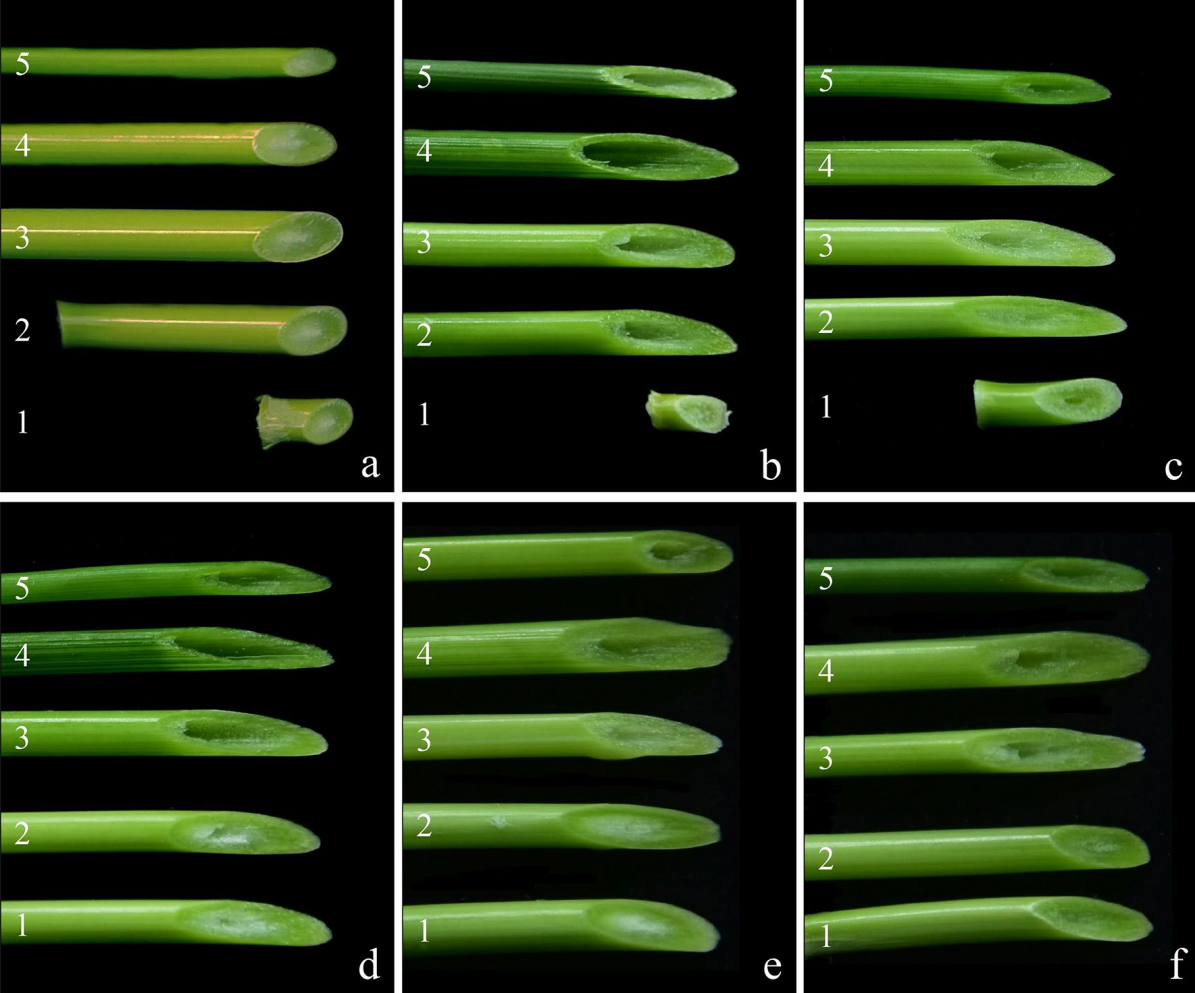
Stalk solidity of synthetic hexaploid wheat grown in the greenhouse. (**a**): Ma; (**b**): CS; (**c**): Syn-SAU-116; (**d**): Syn-SAU-117; (**e**): Syn-SAU-118 and (**f**): Syn-SAU-119. Numbers 1–5 indicate the first to fifth stem internodes (from the base to the top), respectively.

In the greenhouse, the second internode marrow cavity of the stem base of the durum wheat Ma was filled with pith and was considered a solid stem with grade 5.0 (Fig. 4a, Table 2), while that of the common wheat CS had no pith, indicating a hollow stem with grade 1.0 (Fig. 4b, Table 2). The stem solidity of the second internode at the stem base of the four synthetic hexaploid wheat plants was not completely the same. Compared with the common wheat CS, Syn-SAU-116 and Syn-SAU-117 had a marrow cavity and stem wall between the second internodes at the base and were obviously thicker, being considered semisolid stems with grades 4.2 and 3.2, respectively (Fig. 4c, d, Table 2), while Syn-SAU-118 and Syn-SAU-119 were filled with pith in the second internode medullary cavity at the base, exhibiting solid stems with grade 5.0 (Fig. 4e, f, Table 2).

**Figure 5:**
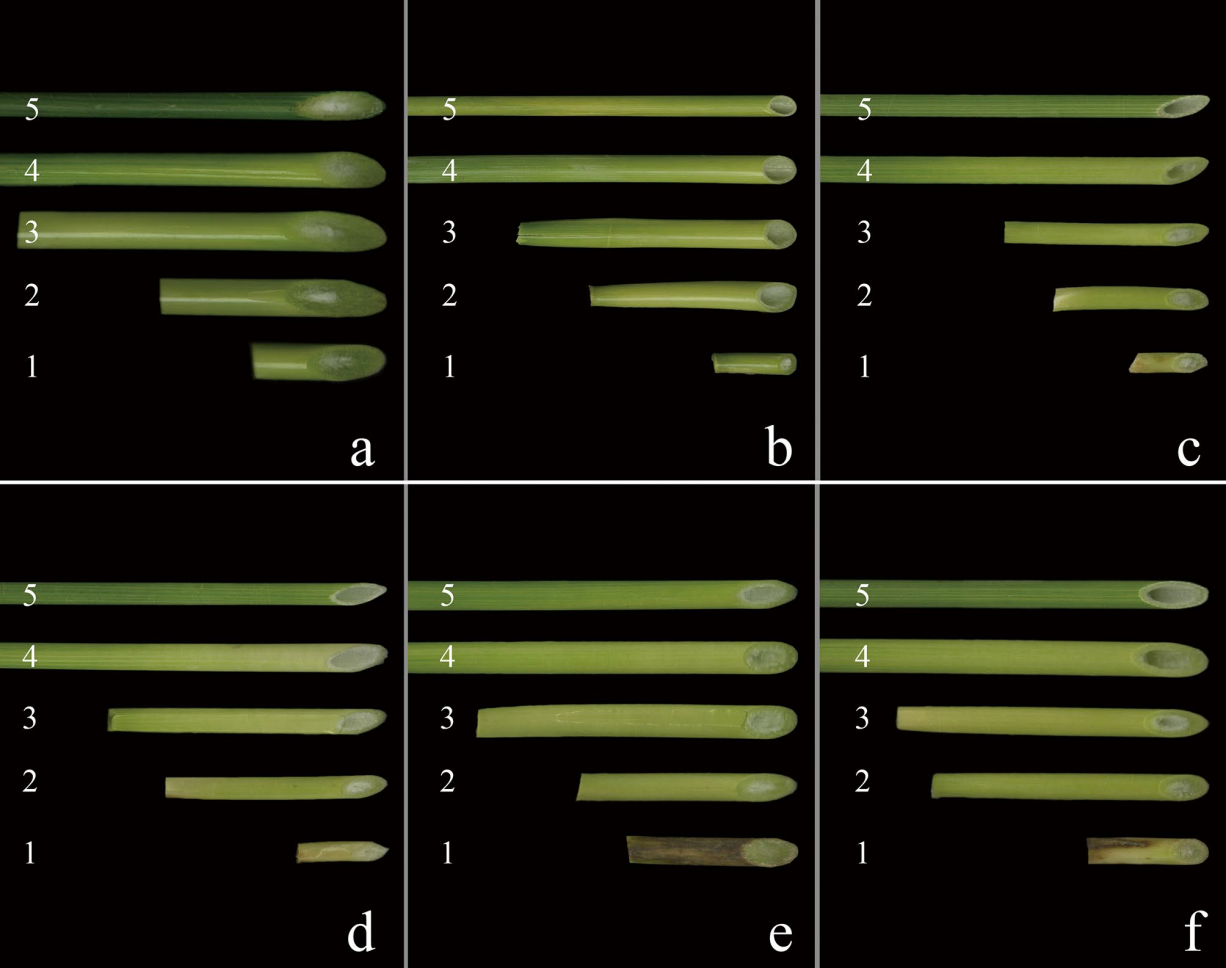
Stalk solidity of synthetic hexaploid wheat grown in the field. (**a**): Ma; (**b**): CS; (**c**): Syn-SAU-116; (**d**): Syn-SAU-117; (**e**): Syn-SAU-118 and (**f**): Syn-SAU-119. Numbers 1–5 indicate the first to fifth stem internodes (from the base to the top), respectively.

In the field, the marrow cavity at the base of the second internode of the durum wheat Ma was also filled with pith, indicating a solid stem with grade 5.0 (Fig. 5a, Table 2), while that of the common wheat CS had no pith, indicating a hollow stem with grade 1.0 (Fig. 5b, Table 2). The stem solidity of the second internode at the base of the four synthetic wheat lines was not completely the same. There was a very small marrow cavity between the second node at the base of Syn-SAU-117 and Syn-SAU-118, indicating semisolid stems with grades 4.1 and 4.5, respectively (Fig. 5d, e), and the stem wall is obviously thicker than that of CS. The medullary cavity of the second intersegment at the base of Syn-SAU-116 and Syn-SAU-119 was filled with pith, indicating a solid stem with grade 5.0 (Fig. 5c, f).

Therefore, the second internode at the base of the stem of Syn-SAU-117 was semisolid with a grade 3.2–4.1, while that of Syn-SAU-119 was solid with grade of 5.0 in both the greenhouse and field.

### Determination of the lodging resistance of synthetic hexaploid wheat grown in the field

The breaking resistance of the second internodes of the stem bases of all four synthetic wheat plants was weaker than that of Ma, and the bending moment was less than that of Ma (Table 3). The breaking resistance, bending moment and lodging index of Syn-SAU-116 were very significant different from that of Ma. The breaking resistance of Syn-SAU-116 was significant different from that of CS, and the bending moment and lodging index were very significant different from that of CS. The breaking resistance of Syn-SAU-117 was significant different from that of Ma, and its lodging index was very significant different from that of CS and Ma. The lodging index of Syn-SAU-118 was very significant different from that of CS. The breaking resistance and of Syn-SAU-119 was significant different from that of Ma, the bending moment was significant different from that of CS and Ma, and the lodging index was very significant different from CS.

**Table 3:** Investigation of lodging resistance of synthetic hexalpoid wheat in the field.

Plant materials	Breaking resistance (N)	Bending moment (cm g)	Lodging index
Chinese Spring	9.068	785.8865	8666.4003
Ma	16.51	890.7525	5395.2302
Syn-SAU-116	7.136*^##^	165.6736**^##^	2321.659**^##^
Syn-SAU-117	9.939^#^	672.5422	6766.4266**^##^
Syn-SAU-118	11.523	700.785	6081.619**
Syn-SAU-119	9.379^#^	474.5922*^#^	5060.2655**

*Significantly different from CS at the 0.05 level, **at the 0.01 level; ^#^ significantly different from *T. durum* Ma at the 0.05 level, ^##^at the 0.01 level.

Compared with Ma, the lodging indices of both Syn-SAU-116 and Syn-SAU-119 were smaller, while the lodging indices of Syn-SAU-117 and Syn-SAU-118 were slightly larger (Table 3). The bending moment and lodging index of the second internodes of the stem bases of the four synthetic wheat plants were lower than those of the CS plants (Table 3). The breaking resistance of the second internode at the base of the stem of Syn-SAU-116 was less than that of CS. The breaking resistance of the second internode at the base of Syn-SAU-117, Syn-SAU-118 and Syn-SAU-119 was greater than that of CS. Syn-SAU-116 had the smallest lodging index, followed by Syn-SAU-119 (Table 3). Lodging resistance was expressed by the lodging index. The smaller the lodging index, the more resistant the plant was. The breaking resistance of synthetic hexaploid wheat was negatively correlated with the lodging index, and the bending moment had a very significant positive correlation with the lodging index (Table 4). Therefore, the synthetic hexaploid wheat Syn-SAU-116 had the strongest lodging resistance, followed by Syn-SAU-119.

**Table 4:** Correlation coefficients between lodging index and mechanical traits in synthetic hexalpoid wheat in the field.

Mechanical traits	Bending moment	Lodging index
Breaking resistance	0.460*	− 0.129
Bending moment		0.798**

* And ** indicate significant at the *P* < 0.05 and *P* < 0.01 levels, respectively.

### The anatomical structure of stalks of synthetic hexaploid wheat grown in the field

**Figure 6:**
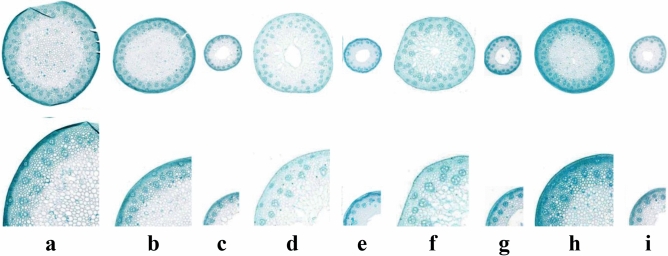
Anatomical structure of the second internode of synthetic hexaploid wheat and its parents in the field. (**a**): Ma; (**b**): Syn-SAU-116; (**c**): AS78; (**d**): Syn-SAU-117; (**e**): AS92; (**f**): Syn-SAU-118; (**g**): AS95; (**h**): Syn-SAU-119; (**i**): AS96.

The outer diameter of the culm of Ma was the largest (Fig. 6a; Table 5). The outer diameters of culms of the *Ae. tauschii* accessions AS78, AS92, AS95, and AS96 were significantly smaller than that of Ma, (Fig. 6c, e, g, i, Table 5). The outer diameters of culms of the four synthetic wheat lines were very significantly different from that of their parents, smaller than that of Ma, but larger than that of their corresponding male parents *Ae. tauschii* (Table 5). Among them, Syn-SAU-116 had the largest outer culm diameter at 4209.18 μm (Fig. 6b, Table 5). However, the width of the pith cavity of Syn-SAU-116 and Syn-SAU-119 was 0 (Table 5), and the pith was full (Fig. 6b, h), the same as that of Ma. Syn-SAU-117 had the largest widths of pith cavities, twofold larger than Syn-SAU-118 (Fig. 6d, f, Table 5), different from that of Ma. The width of the pith cavity of Syn-SAU-117 and Syn-SAU-118 were very significantly different from Ma. The width of the pith cavity of four synthetic wheat lines were all very significantly different from their corresponding male parents.

**Table 5:** Comparisons of stem character of synthetic hexalpoid wheat and their parents in the field.

Plant materials	Outer diameter of culm (μm)	Width of pith cavity (μm)	Ratio of wall thickness to outer diameter of culm (%)	Percentage of mechanical tissues (%)	No. vascular bundles in transverse section
Ma	4669.54	0	50	25.99	64
Syn-SAU-116	4209.18**^##^	0^##^	50^##^	28.27**^##^	59**^##^
AS78	1471.58	580.93	30.26	14.25	27
Syn-SAU-117	3563.52**^##^	709.27**^##^	40.05**	21.48**	62.33**^##^
AS92	1509.86	287.95	40.46	21.39	40
Syn-SAU-118	3380.75**^##^	324.76**^##^	45.2**^##^	26.38^##^	52**^##^
AS95	1692.28	721.06	28.7	13.52	30
Syn-SAU-119	3557.54**^##^	0^##^	50^##^	34.29**	62.5**^##^
AS96	1726.55	523.32	34.85	31.67	38

*Significantly different from *T. durum* Ma at the 0.05 level, **at the 0.01 level; ^#^ significantly different from *Ae. tauschii* at the 0.05 level, ^##^at the 0.01 level.

Syn-SAU-116 and Syn-SAU-119 had the largest ratio of wall thickness to outer culm diameter, reaching 50%, the same as that of Ma (Table 5). There was very significantly different between Syn-SAU-116, Syn-SAU-119 and their corresponding male parents. The ratio of wall thickness to outer culm diameter of Syn-SAU-117 was very significantly different from that of Ma. The ratio of wall thickness to outer culm diameter of Syn-SAU-118 was very significantly different from that of its parents.

Syn-SAU-116, Syn-SAU-118 and Syn-SAU-119 had a larger percentage of mechanical tissue than Ma. Among them, Syn-SAU-119 had the largest percentage of mechanical tissue with 34.29%, and Syn-SAU-117 had a slightly smaller percentage of mechanical tissue than Ma with 21.48% (Table 5). The percentage of mechanical tissue of Syn-SAU-116 was very significantly different from that of its parents (Table 5). The percentage of mechanical tissue of Syn-SAU-117 and Syn-SAU-119 were very significantly different from Ma. The percentage of mechanical tissue of Syn-SAU-118 was very significantly different from its male parents.

Ma had a large number of vascular bundles, as many as 64, while that of four *Ae. tauschii* accessions was 27–40, much less than that of Ma (Table 5). The number of vascular bundles of the four synthetic wheat plants was 52–62.5, less than that of Ma (Table 5), but much larger than that of their corresponding male parents. There were very significantly different in vascular bundles between four synthetic hexaploid wheats and their parents.

In this study, it was indicated that the width of the pith cavity had a very significant positive correlation with the lodging index for synthetic hexaploid wheat (Table 6). The percentage of mechanical tissue had a negative correlation with the lodging index. The outer diameter of the culm of the second internode at the base of the stem had a significant negative correlation with the lodging index. The ratio of wall thickness to the outer culm diameter had a very significant negative correlation with the lodging index. There was no correlation between the number of vascular bundles and the lodging index.

**Table 6:** Correlation coefficients between lodging index and traits in synthetic hexalpoid wheat in the field.

Traits	Width of pith	Ratio of wall thickness to outer diameter of culm	Percentage of mechanical tissues	Number of vascular bundles in transverse section	Lodging index
Outer diameter of culm	− 0.28	0.306	− 0.119	0.402	− 0.539*
Width of pith cavity		− 0.998**	− 0.699**	0.129	0.677**
Ratio of wall thickness to outer diameter of culm			0.694**	− 0.09	− 0.686**
Percentage of mechanical tissues				− 0.018	− 0.326
No. vascular bundles in transverse section					0.078

*And ** indicate significant at the *P* < 0.05 and *P* < 0.01 levels, respectively.

### Chromosomal observations of four synthetic hexaploid wheat lines

**Figure 7:**
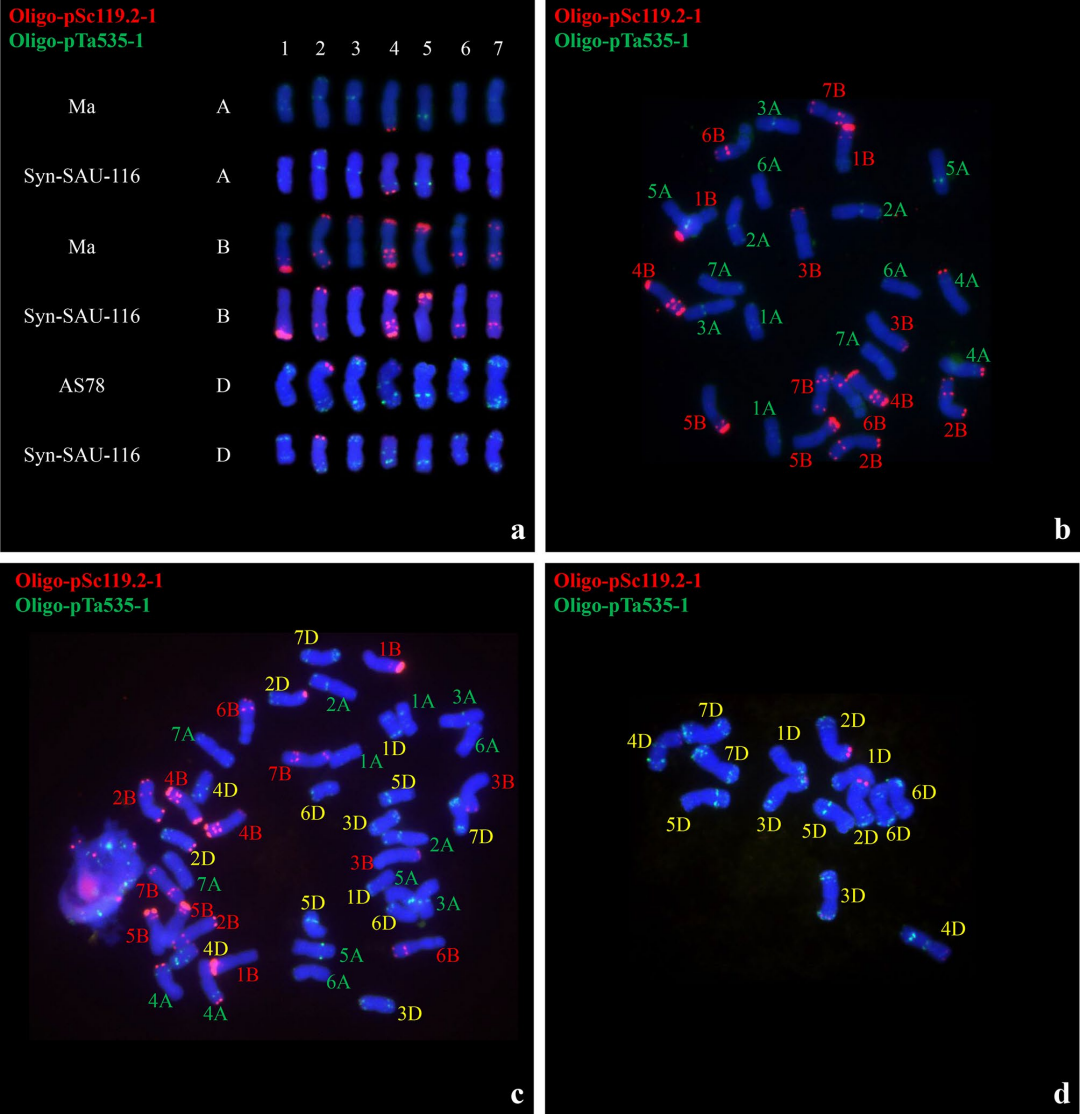
FISH identification of Syn-SAU-116 and its parent. (**a**): FISH karyotypes of the A, B, and D genomes in Syn-SAU-116 and its parents; (**b**): Ma; (**c**): Syn-SAU-116; (**d**): AS78.

**Figure 8:**
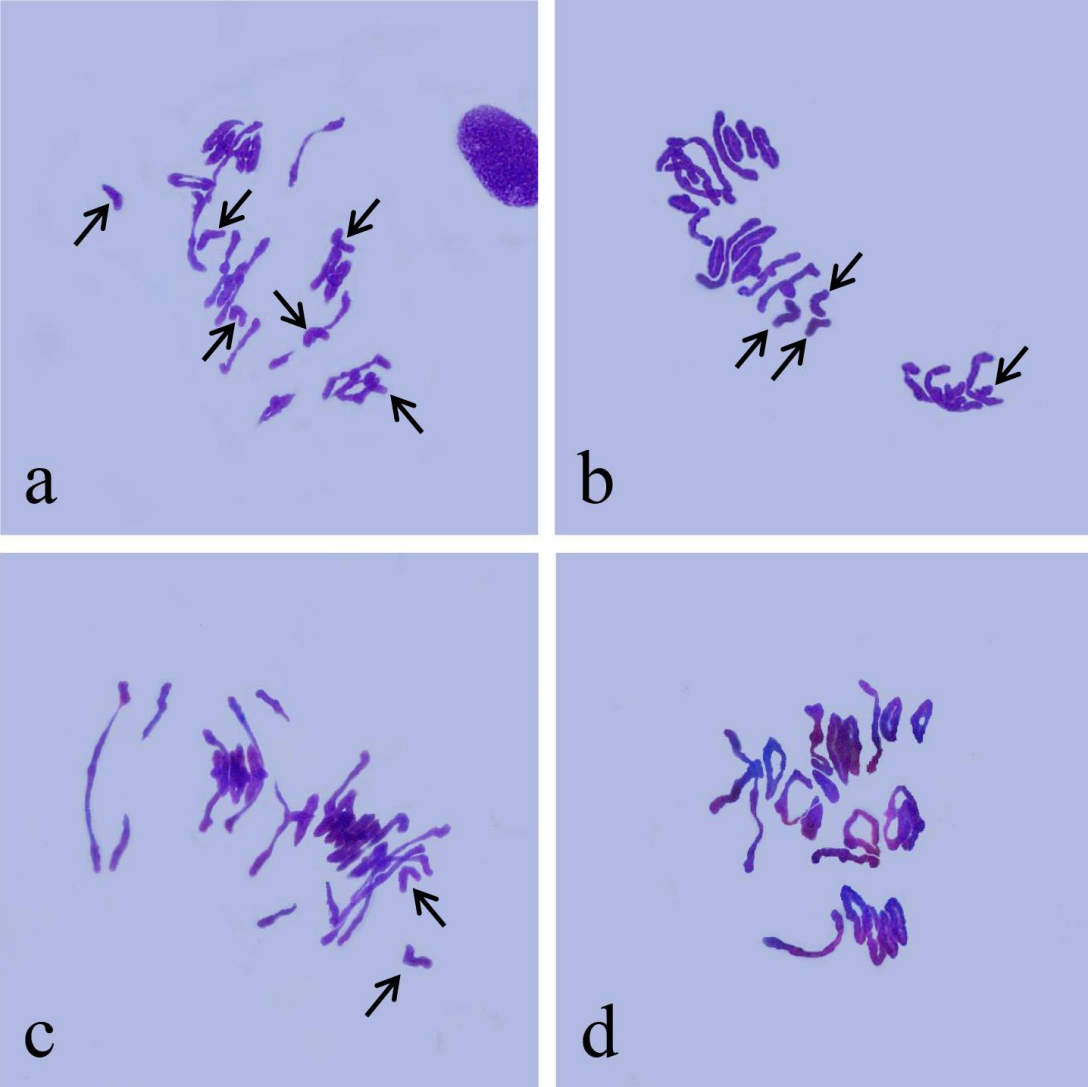
Chromosome pairings of pollen mother cells at meiotic metaphase I in synthetic hexaploid wheat. (**a**): Syn-SAU-116 (6I + 18II); (**b**): Syn-SAU-117 (4I + 19II); (**c**): Syn-SAU-118 (2I + 20II); (**d**): Syn-SAU-119 (21II). Arrows indicate univalents.

Analysis of root tip chromosome numbers showed that of 47 plants from four synthetic hexaploid wheat lines, 32 had 42 chromosomes, while 15 had 41 chromosomes (Table 7). Multicolor FISH was performed on the plants of four synthetic hexaploid wheat lines with 42 chromosomes using probes Oligo-pTa535-1 and Oligo-pSc119.2-1 (Fig. 7, Supplementary Information). The A-, B-, and D-genome chromosomes were distinguished according to Tang et al.^48^. The green-labeled Oligo-pTa535-1 probe mainly hybridized to the A- and D-genome chromosomes (Fig. 7, Supplementary Information). The red-labeled Oligo-pSc119.2-1 probe mainly hybridized to the B-genome chromosome, along with the signals at the end of the long arm of 4A and the end of the short arm of 2D, 3D and 4D (Fig. 7, Supplementary Information). Plants with 42 chromosomes were selected for the observation of chromosome pairing of PMCs in meiotic metaphase I. Most of the 42 chromosomes paired as bivalents (Fig. 8, Table 7), while a low number of univalent PMCs were also observed, indicating relative cytological stability.

**Table 7:** Chromosome observation of synthetic hexalpoid wheat.

Code	No. of plants observed		Chromosome pairing configuration of synthetic hexaploid wheat
	n = 41	n = 42	
Syn-SAU-116	6	6	5.92 I + 10.56 rod II + 7.48 ring II
Syn-SAU-117	5	11	5.38 I + 12.67 rod II + 5.64 ring II
Syn-SAU-118	2	9	4.32 I + 10.06 rod II + 8.78 ring II
Syn-SAU-119	2	6	6.08 I + 8.61 rod II + 9.35 ring II

## Discussion

Lodging remains a problem in wheat-growing regions worldwide, although scientists have made great efforts over many years. The selection of elite accessions with alternative semidwarfing alleles or high stem mechanical strength may be a powerful approach to reducing this problem^11^. Durum has an abundance of solid-stemmed varieties, landraces and old varieties^24^. Although the solid stem of one durum wheat line, Golden Ball, has been transferred into a common wheat AC Elsa background through one synthetic hexaploid wheat, P89-77-1F_4_^41^, two solid-stemmed derivatives of P89-77-1F_4_ were still taller and later maturing than AC Elsa, which averaged 95 cm and reached maturity in 104 d in the brown soil zones and in 107 d in the dark brown soil zones^40^. Thus, it is important to transfer solid stems from more durum wheat lines to hexaploid wheat.

In this study, four new synthetic hexaploid wheat lines with solid stems were developed and identified, which were different from the reported synthetic hexaploid wheat P89-77-1F_4_ based on their different pedigrees. Moreover, these new synthetic hexaploid wheat lines are shorter than some reported synthetic hexaploid wheat lines^51,52^. The four solid-stem synthetic wheat plants simultaneously carry both the genetic material of *T. durum* and *Ae. tauschii*, which is different from solid-stem wheat such as Xiaoyan 81, 86–741, XSXS, WYSG, etc.^12,13,53,54^. In this study, the expression of a solid second internode at the base of the stem was stable for two synthetic hexaploid wheat lines, Syn-SAU-117 and Syn-SAU-119, grown in both the greenhouse and field. The second internode at the base of the stem of Syn-SAU-117 was semisolid, while that of Syn-SAU-119 was solid in both the greenhouse and field. This difference may have been caused by the different male parents, which were all *Ae. tauschii*. There may be suppressor genes on the chromosomes of the D genome in *Ae. tauschii* AS92 to suppress the solid expression of stems in Syn-SAU-117, but this needs to be further studied.

Previous studies have shown that crop lodging resistance is closely related to plant height, internode length, internode thickness, internode wall thickness, and internode fullness^12,17^. More vascular bundles, larger vascular bundle areas, and thicker mechanical tissue and parenchyma are all conducive to the improvement of lodging resistance^7,17^. In this study, all four synthetic wheat samples had large outer diameters, very small or no pith cavity, well-developed mechanical tissues, thick stalk walls and a large number of vascular bundles in the second internodes of the base. These lines showed strong lodging resistance, which was in agreement with the selection characteristics of modern cereal crops for lodging resistance breeding^16^. The lodging resistance of the four synthetic solid-stem wheat samples was stronger than that of CS, and Syn-SAU-116 had the strongest lodging resistance, followed by Syn-SAU-119.

Stripe rust is one of the most serious biological stresses in global wheat production. In this study, these four synthetic hexaploid wheat varieties had high resistance to stripe rust, which will provide new resistant sources for wheat improvement. These synthetic hexaploid wheat lines can be used as "bridges" to introduce solid-stemmed traits into common wheat for lodging resistance improvement. The work is ongoing to transfer solid stems to common wheat cultivars by crossing these solid-stemmed synthetic hexaploid wheat lines with elite common wheat varieties following Ref^52^.

## Conclusions

Four new synthetic hexaploid wheat lines with solid stems were developed and identified by molecular cytogenetic method. The solid expression of the second internode at the base of the stem was stable for two synthetic hexalpoid wheats Syn-SAU-117 and Syn-SAU-119 grown in both the greenhouse and field. Syn-SAU-116 has the strongest lodging resistance, followed by Syn-SAU-119. Four synthetic wheat lines had large outer diameters, well-developed mechanical tissues, a large number of vascular bundles, and anatomical characteristics. At the adult stage, all four synthetic hexaploid wheat lines showed high resistance to mixed physiological races of the stripe rust pathogen (CYR31, CYR32, CYR33, CYR34). These synthetic hexaploid wheat lines provide new materials for the improvement of common wheat.

## Supplementary Information

Supplementary Information.
