# Supplementary material for: Development and identification of four new synthetic hexaploid wheat lines with solid stems
Source: Sci Rep. 2022 Mar 22;12:4898. doi: 10.1038/s41598-022-08866-x (PMC8941074; doi:10.1038/s41598-022-08866-x)
Supplement: Supplementary file 1 — Supplementary Information. [file 41598_2022_8866_MOESM1_ESM.pdf]

Development and identification of four new synthetic hexaploid wheat lines with solid stems

Dongyu Liang[2], Minghu Zhang[2], Xin Liu[2], Hui Li[2], Zhenjiao Jia[2], Dinghao Wang[2], Ting Peng[2], Ming Hao[2], Dengcai Liu[1,2], Bo Jiang[2], Lin Huang[1,2], Shunzong Ning[2], Zhongwei Yuan[2], Xuejiao Chen[2], Lianquan Zhang[1,2]*

[1] *State Key Laboratory of Crop Gene Exploration and Utilization in Southwest China, Sichuan Agricultural University, Wenjiang, Chengdu, 611130, China*

[2] *Triticeae Research Institute, Sichuan Agricultural University, Wenjiang, Chengdu, 611130, China*

Supplementary Information

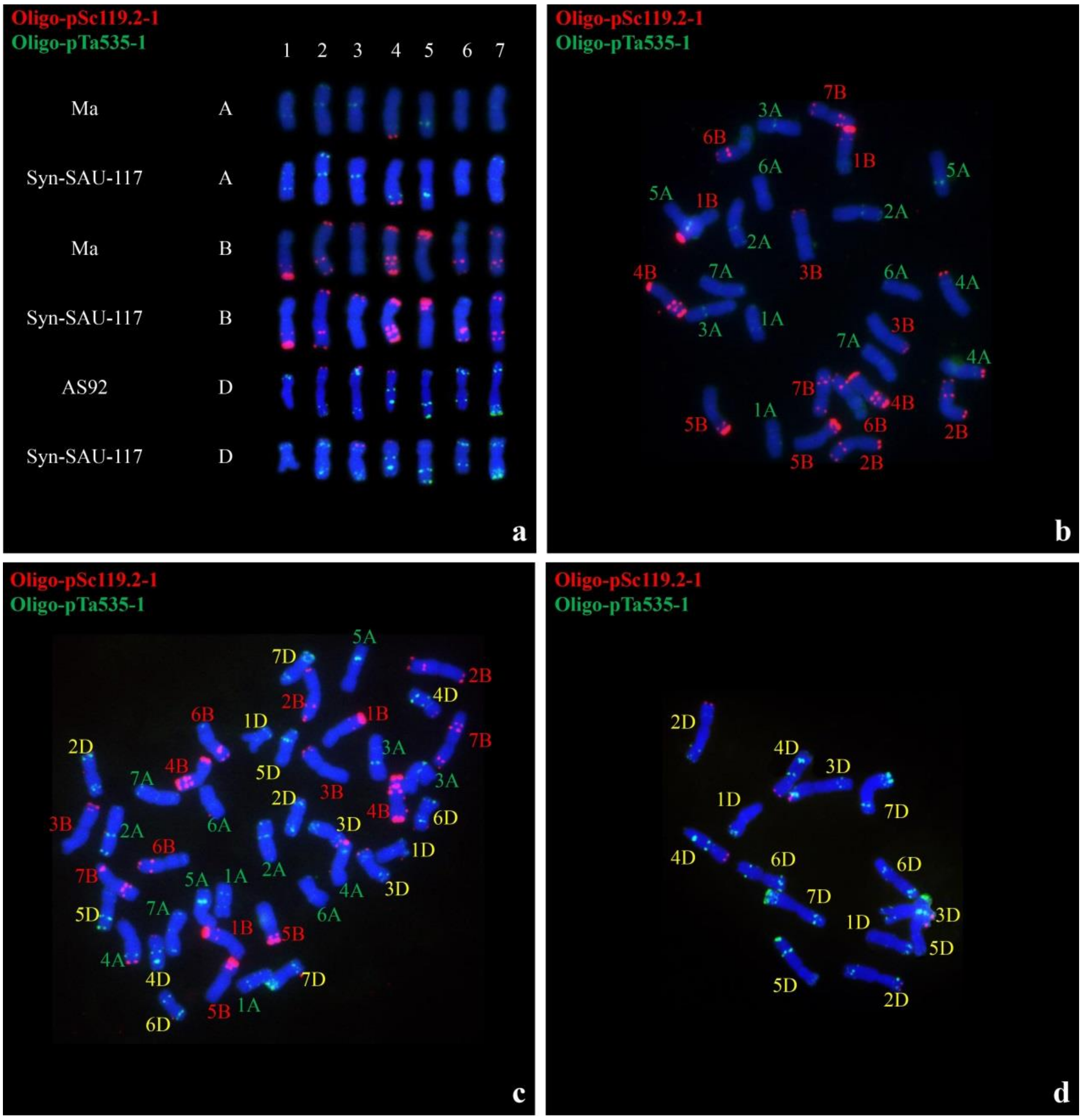

Supplementary Figure S1 – FISH identification of Syn-SAU-117 and its parent. a: FISH karyotypes of the A, B, and D genomes in Syn-SAU-117 and its parents; b: Ma; c: Syn-SAU-117; d: AS92.

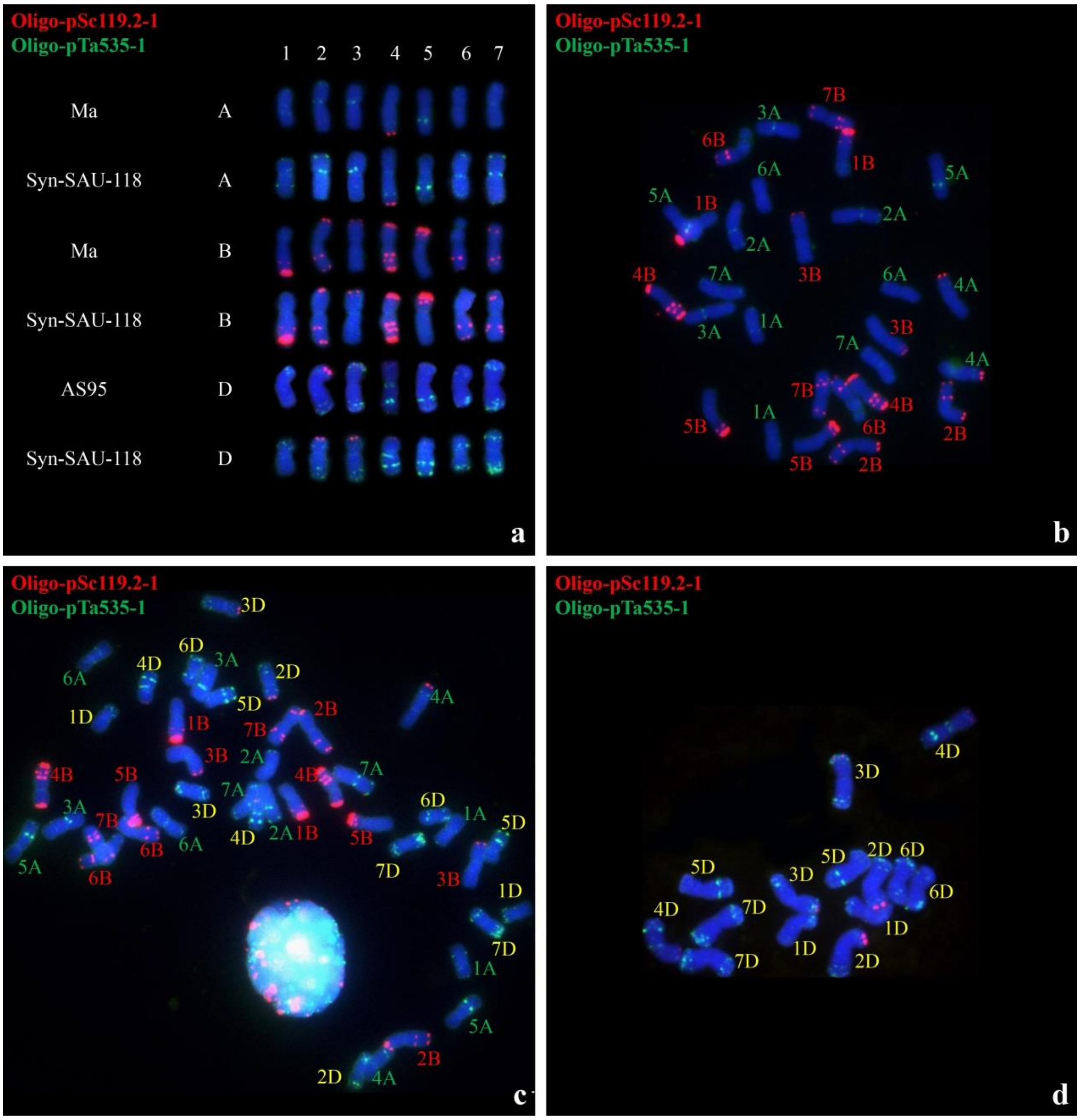

Supplementary Figure S2 – FISH identification of Syn-SAU-118 and its parent. a: FISH karyotypes of the A, B, and D genomes in Syn-SAU-118 and its parents; b: Ma; c: Syn-SAU-118; d: AS95.

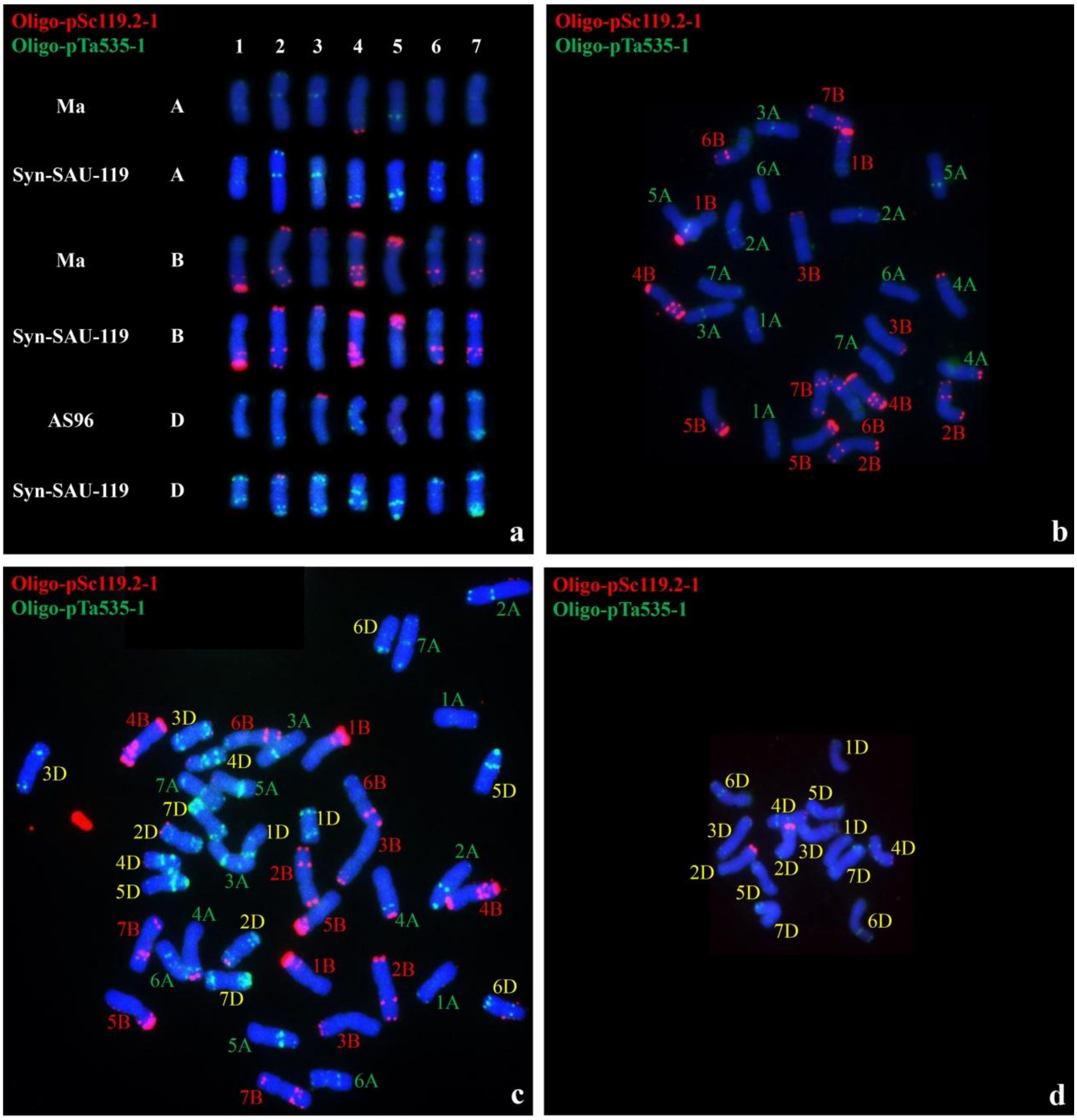

Supplementary Figure S3 – FISH identification of Syn-SAU-119 and its parent. a: FISH karyotypes of the A, B, and D genomes in Syn-SAU-119 and its parents; b: Ma; c: Syn-SAU-119; d: AS96.
